# Hyperbaric Oxygen Therapy Is Associated with Lower Mortality in Patients with Fournier’s Gangrene: A Meta-Analysis

**DOI:** 10.3390/medicina62061199

**Published:** 2026-06-22

**Authors:** Chienhsiu Huang, Lichen Lin, Tiju Tang

**Affiliations:** Department of Internal Medicine, Dalin Tzu Chi Hospital, Buddhist Tzu Chi Medical Foundation, NO. 2, Min-Sheng Road, Dalin Town, Chiayi County 622401, Taiwan

**Keywords:** Fournier’s gangrene, hyperbaric oxygen therapy, mortality, surgical debridement, broad spectrum antibiotic

## Abstract

*Background and Objectives*: The therapeutic management of Fournier’s gangrene involves the use of broad-spectrum antibiotics and adequate surgical debridement; however, the overall mortality rate remains high globally. Hyperbaric oxygen therapy may serve as an adjunct treatment modality for reducing mortality in patients with Fournier’s gangrene, but its efficacy remains debatable. Consequently, this meta-analysis assessed the effect of hyperbaric oxygen therapy on mortality in patients with Fournier’s gangrene. *Materials and Methods*: To assess the effect of hyperbaric oxygen therapy on patients with Fournier’s gangrene, various databases were meticulously searched by focusing on the study population, intervention, control, and mortality outcomes. *Results*: Fourteen studies, enrolling a total of 793 patients, including 356 in the hyperbaric oxygen therapy cohort and 437 in the nonhyperbaric oxygen therapy cohort, were included in this meta-analysis. Compared with the mortality rate of 27.45% in the control cohort, the rate in the hyperbaric oxygen therapy cohort was 9.55%. This difference between patients with Fournier’s gangrene who underwent conventional therapy with hyperbaric oxygen therapy and those receiving only conventional therapy was significant (RR = 0.33; 95% CI 0.23–0.48; *p* < 0.001). *Conclusions*: Hyperbaric oxygen therapy may serve as an adjunct intervention to mitigate the increased risk of mortality among patients with Fournier’s gangrene. Methodologically rigorous randomized controlled trials must be conducted to determine the appropriate dosage and therapeutic efficacy of hyperbaric oxygen therapy in these patients and identify specific patient cohorts poised to obtain a benefit from this treatment.

## 1. Introduction

Fournier’s gangrene (FG) is an uncommon, swiftly advancing necrotizing fasciitis of the soft tissue that affects scrotal, perineal, anal, and genital anatomical regions. FG can affect both sexes and individuals of various age groups; however, it occurs more frequently in males than in females and in individuals aged more than 50 years. Diabetes mellitus has emerged as the most prevalent comorbidity, while other common comorbidities include obesity, cardiovascular disease, chronic kidney disease, liver disease, malignancy, and alcoholism [1,2,3]. The diagnosis of FG can be challenging and is often delayed or missed because of its variable severity of presentation and its propensity to involve deeper tissues and fascia while sparing the overlying skin and superficial tissues during the early stages of the disease. As many as 40% of patients diagnosed with FG may initially present without any dermatological manifestations, which increases the likelihood of diagnostic delay and advancement to a condition associated with elevated mortality rates [4,5].

The management of FG is a multifaceted challenge that requires an interdisciplinary methodology. It is predicated on three fundamental interventions: prompt surgical debridement, empirical administration of broad-spectrum antibiotics, and intensive supportive care [6]. From a microbiological perspective, FG is invariably characterized as a polymicrobial infection that encompasses both aerobic and anaerobic organisms [7]. Synergistic interactions among these microbes substantially exacerbate tissue necrosis. Consequently, the timely collection of cultures and the rapid commencement of broad-spectrum empirical antibiotic therapy, followed by targeted treatment strategies, are essential elements of FG management [8].

The two main prognostic scoring scales are specific and exhibit good reliability in assessing FG severity and mortality risk. The Fournier’s gangrene scoring index (FGSI), which was developed by Laor et al., has been widely used to analyse clinical signs and laboratory markers to predict patient outcomes and [9]. This system was expanded by Yılmazlar et al. into Uludag FGSI [10].

In 2000, Eke et al. reported 1726 FG cases with a mortality rate of 16% [11]. In 2016, Sorensen et al. reported 1641 FG cases in the U.S. with a mortality rate of 7.5% [12]. However, the global mortality of FG has remained high, ranging from 20% to 40% [13,14]. The reported mortality rates are variable because of discrepancies in the characterization of patient populations, microbial aetiological factors, and the implementation of therapeutic interventions. Shet al. thoroughly analysed the risk factors for mortality in patients with FG and reported a mortality rate of 20.4% among a cohort of 3646 patients. Female sex, presence of comorbidities, anatomical distribution, development of sepsis, and fungal infections have been shown to increase the risk of mortality [15]. An increased need for operations and repeated surgeries was also associated with a higher mortality risk [16].

FG continues to be associated with elevated mortality rates because of inadequate local blood circulation in patients, which leads to infection and vascular impairment, thereby prolonging the healing process [17]. Identifying an adjunct therapeutic approach to conventional treatment modalities is imperative, as it may substantially increase survival rates and decrease the mortality risk in patients with necrotizing fasciitis. Hyperbaric oxygen therapy (HBOT) represents a therapeutic intervention that involves inhaling pressurized 100% oxygen in a sealed chamber operating at pressures exceeding atmospheric levels.

In the context of necrotizing soft tissue infections, HBOT has been postulated to enhance the effective diffusion range of oxygen, thereby preserving critically compromised ischaemic regions; facilitate the eradication of bacteria through direct actions on anaerobic pathogens via the production of oxygen free radicals, as well as the increase in the phagocytic function of leukocytes; increase the efficacy of antibiotics such as beta-lactams, aminoglycosides, and quinolones, whose action mechanisms are partially dependent on bacterial aerobic metabolism; the effectiveness of vancomycin, teicoplanin, and linezolid is notably increased when these drugs are administered concurrently with HBOT for infections caused by methicillin-resistant *Staphylococcus aureus*; promote fibroblast proliferation, stimulate collagen synthesis, and increase angiogenesis, thereby facilitating wound healing; additionally, exert anti-inflammatory effects, mitigating endothelial injury, reducing ischaemia–reperfusion–induced damage, decreasing inflammatory cytokine levels, and increasing the synthesis of anti-inflammatory enzymes [18,19,20,21].

Thus, HBOT has been used as an adjunct treatment modality for FG; however, the specific indications and its effect on mortality outcomes remain a subject of debate [22]. Several studies have reported that HBOT contributes to a significant reduction in mortality among patients with FG [1]. The 2024 European Association of Urology Guidelines on Urological Infections recommend that adjunct treatments for FG be administered only within the framework of clinical trials [23]. A consensus on the efficacy of adjunct therapy with HBOT in patients with FG is lacking, particularly in relation to its potential to reduce mortality among affected patients [24]. Therefore, the purpose of this meta-analysis was to investigate the effect of HBOT on mortality in FG patients.

## 2. Materials and Methods

### 2.1. Search Strategy and Selection Criteria

Using the primary search keywords “Fournier gangrene”, “penile necrotizing fasciitis”, “hyperbaric oxygen”, “hyperbaric oxygen therapy”, and “hyperbaric oxygen treatment”, we searched electronic databases, such as PubMed, the Cochrane Library, and Web of Science, from 1 January 1990 until 28 February 2026 to find pertinent published trials and meta-analyses or systematic reviews on the topic. Previously published systematic reviews and meta-analyses were reviewed to identify any additional studies that may have been missed in the primary literature search. No restrictions were applied on the use of English.

### 2.2. Inclusion and Exclusion Criteria

Articles eligible for inclusion were randomized controlled trials or observational studies comprising two groups of patients and reporting mortality rates in patients with FG treated with conventional therapy with HBOT and conventional therapy alone without HBOT. Each group was required to include at least five patients, or both groups of participants were required to include at least twenty cases. Experimental studies conducted on animal models, unpublished manuscripts, and findings reported solely in the abstract form were excluded from the analysis. HBOT is an adjunct treatment modality in which patients inhale 100% oxygen under increased atmospheric pressure.

### 2.3. Data Extraction

Two autonomous researchers collected the data in accordance with the predefined extraction template. Any inconsistencies or conflicts pertaining to data extraction were discussed and resolved by a third investigator if necessary. The extracted items included the authors, year of publication, study design, number of participants in each treatment group, and mortality rate, all of which were derived from studies that met the established inclusion criteria. We also explored the risk factors for mortality among patients with FG receiving HBOT.

### 2.4. Risk of Bias Assessment and Statistical Analysis

The Risk of Bias in Nonrandomized Studies of Interventions (ROBINS-I) tool was used to evaluate observational studies [25]. Data were entered into the Cochrane Review Manager software RevMan 5.4. Differences are expressed as risk ratios (RRs) with 95% confidence intervals (CIs) for dichotomous outcomes. The significance of the pooled ratios was determined using the Z test, and a *p* value of less than 0.05 was considered to indicate statistical significance. Cochran’s Q and I^2^ tests were used to assess the heterogeneity of the data included in each outcome. A significance level of *p* < 0.1 was considered for the Q statistic. To account for the limited number of studies included in many of the analysed outcomes, I^2^ values above 50% were considered to indicate significant heterogeneity. A fixed-effects model was used when the effects were assumed to be homogenous, whereas a random-effects model was used when the effects were heterogeneous. Publication bias was assessed by examining funnel plots. Furthermore, Fisher’s exact test was employed to investigate risk factors for mortality among FG patients receiving HBOT.

This review was performed in accordance with the PRISMA (Preferred Reporting Items for Systematic Reviews and Meta-Analyses) guidelines. The Systematic Review and Meta-Analysis was registered at the Prospero international prospective register of systematic reviews (registration date: 14 April 2026) (registration ID: CRD420261369219).

## 3. Results

**Figure 1:**
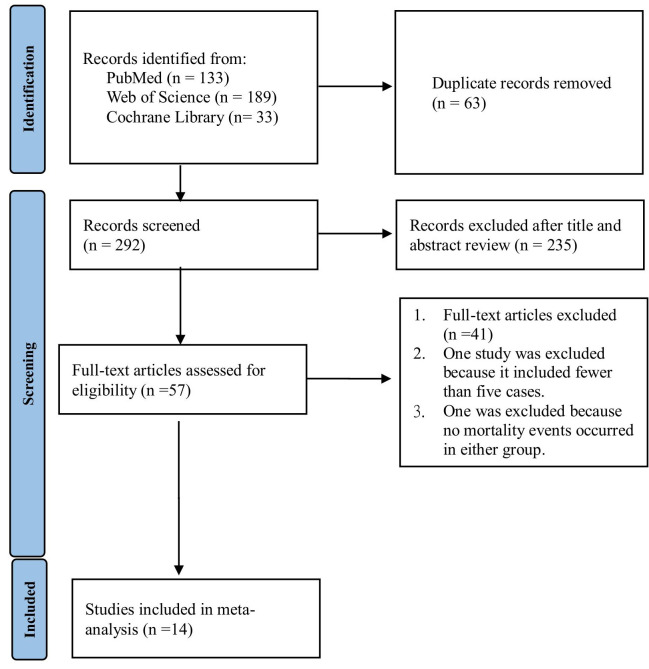
Flow diagram of the study selection process.

**Figure 2:**
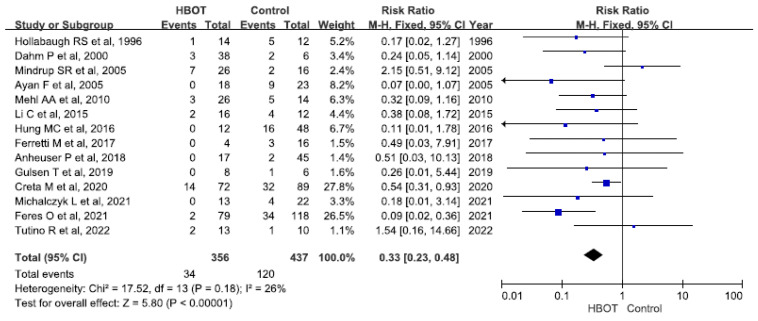
Forest plot for the mortality rate of FG patients in HBOT and non-HBOT groups [22,28,29,30,31,32,33,34,35,36,37,38,39,40].

**Figure 3:**
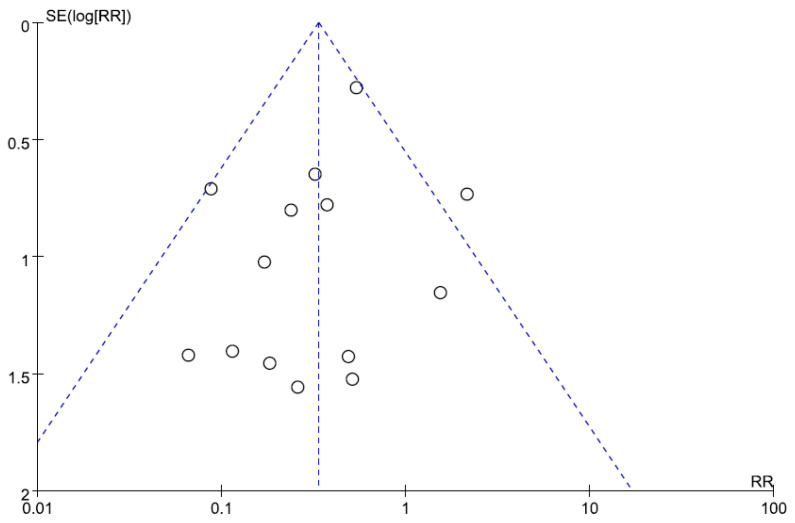
Funnel plots for the mortality rate of FG patients in HBOT and non-HBOT groups.

The details of the study selection process are shown in Figure 1. The numbers of studies obtained from the initial searches of PubMed, Web of Science, and the Cochrane Library were 133, 189, and 33, respectively. A total of 63 articles were duplicates. A total of 235 irrelevant studies were identified by reading the title and/or abstract. After duplicates and irrelevant studies were excluded, 57 potentially relevant articles remained. After the full-text review was completed, 41 articles were excluded because they did not report mortality rates in patients with FG treated with conventional therapy with HBOT compared with patients treated with conventional therapy alone. One study was excluded because it included fewer than five patients [26], and another was excluded because no mortality events occurred in either group [27]. Finally, 14 studies were included in the meta-analysis [22,28,29,30,31,32,33,34,35,36,37,38,39,40]. The main characteristics of the included studies are shown in Table 1. Baseline differences in clinical severity, comorbidities, disease severity, or the HBOT protocol were observed among the 14 studies. All the studies had a high risk of bias (Table 2). Ten studies reported risk factors for mortality among patients with FG who underwent HBOT (Table 3) [27,28,33,40,41,42,43,44,45,46]. A total of 793 patients were analysed in this meta-analysis, with 356 in the HBOT group and 437 in the non-HBOT group. The intervention groups in each study received different doses of HBOT. The overall mortality rate in the HBOT group was 9.55%, whereas that in the control group was 27.45%. A significant difference in mortality was observed between patients who received conventional therapy with HBOT and those who received only conventional therapy alone (RR = 0.33; 95% CI 0.23–0.48; *p* < 0.001) (Figure 2). Figure 2 shows an I^2^ value of 26%, which indicates low to moderate heterogeneity among the included studies, Cochran’s Q (Chi^2^) of 17.52, degrees of freedom (df) of 13, and the *p* value for heterogeneity of 0.18, which is not statistically significant and indicates no significant between-study heterogeneity. The funnel plot (Figure 3) appeared reasonably symmetrical around the pooled effect size. No obvious absence of studies was observed on the left or right side of the funnel. The funnel plot demonstrated no obvious visual evidence of substantial publication bias for mortality outcomes in patients with FG receiving HBOT versus non-HBOT treatment.

## 4. Discussion

### 4.1. HBOT Reduces Mortality in Patients with FG

HBOT accelerates the healing process and has a bactericidal effect on infections caused by aerobic or anaerobic bacteria. However, the optimal duration and frequency of treatment for necrotizing soft tissue infections remain to be definitively determined, and standardized protocols are lacking. The 10th European Consensus Conference (2017) on Hyperbaric Medicine established the following consensus-based recommendations regarding standard practices in hyperbaric medicine: 1. HBOT is advocated for the management of anaerobic or mixed bacterial infections; 2. HBOT should be employed as a treatment for necrotizing soft tissue infections in any anatomical location, with a particular emphasis on FG; and 3. HBOT must be integrated with prompt surgical intervention and broad-spectrum antibiotic therapy tailored to address prevalent anaerobic and aerobic bacterial pathogens [47].

Some studies have reported that HBOT did not reduce mortality in patients with FG. Mindrup et al. observed that the HBOT group had a higher mortality rate (26.9%) than the non-HBOT group (12.5%) [30]. This increase in mortality may be attributable to selection bias, as more severely ill patients may have required HBOT. In addition, the median follow-up period in this study was 4.2 years, which was significantly longer than that in other studies. The severity of FG and longer follow-up duration are important factors for determining the real outcomes and prognosis of patients with FG. In Tutino et al.’s study, a mortality rate of 15.38% was reported in the hyperbaric oxygen group compared with 10.0% in the conventional therapy group, indicating that HBOT did not improve the survival of patients with FG [40]. A higher percentage of female patients with FG (7/23; 30.4%) was included in this study, including two female patients who died after receiving HBOT. Shet P et al. reported higher mortality rates among females (29.76%) than among males (20.48%) [15]. The higher percentage of female patients with FG may explain why HBOT did not increase the survival rate of patients with FG in Tutino’s study.

Several meta-analyses and systematic reviews have investigated the effect of HBOT on patients with FG and have shown markedly lower mortality rates in the HBOT group than in the non-HBOT group. In their comprehensive systematic review, Schneidewind L et al. included five studies that collectively involved 319 patients diagnosed with FG, of whom 145 were administered HBOT. A mortality rate of 16.6% was observed in the HBOT cohort and 25.9% in the non-HBOT cohort [8]. In their systematic review, Shet P. et al. included 57 studies, comprising 3646 patients with FG, with an overall mortality rate of 20.41%. Among these patients, 42 patients received HBOT, for whom the mortality rate was 11.9% [15]. A meta-analysis included 10 retrospective studies published from 1998 to 2021 and involved 269 patients who received HBOT [48]. The HBOT group had a significantly lower mortality rate than the non-HBOT group (odds ratio 0.29, *p* = 0.005) [48]. In their meta-analysis, Patel A et al. (2026) [49] included 9 retrospective studies and 4 case series, with 229 patients receiving HBOT. The HBOT group had a significantly lower mortality rate (11.5%) than the non-HBOT group (26.0%) (RR = 0.43, 95% CI 0.21–0.86). The authors suggested that HBOT reduces mortality in patients with FG and could be considered an adjunct therapy. An umbrella review by Müssgens C et al. (2026) included seven systematic review articles and concluded that HBOT may reduce mortality, although the evidence remains limited [50]. The primary objective of this review was to assess the effect of HBOT on mortality in patients with FG. This meta-analysis included 14 retrospective studies published from January 1990 to February 2026, in which 356 patients received HBOT. Our findings revealed a significant reduction in mortality among patients receiving conventional therapy with HBOT compared with those receiving conventional therapy alone. Compared with the 27.45% mortality rate in the control group, the overall mortality rate in the HBOT group was 9.55%. The meta-analysis revealed a relative risk of mortality of 0.33, indicating that patients treated with HBOT had a 67% lower risk of death than patients receiving the control treatment did.

### 4.2. Risk Factors for Mortality in Patients with FG Receiving HBOT

In their systematic review, Shet P. et al. explored risk factors for mortality among patients with FG and revealed that the mean FGSI score for nonsurvivors was 10.09 compared with 4.33 for survivors, with studies consistently showing that a cut-off score of nine or more was strongly associated with mortality (OR = 4.11, *p* = 0.024). The mean age of patients who died was 61.27 years, whereas that of survivors was 53.21 years. The mortality rate was noticeably higher among females (29.76%) than among males (20.48%) [15]. In the current meta-analysis, two studies reported FGSI scores for 22 patients who received HBOT. No deaths occurred among patients with an FGSI score ≤ 9, whereas 2 patients (2/7; 28.5%) with an FGSI score greater than 9 died. However, the difference between these groups was not significant (*p* = 0.091). Nine studies reported the sex distribution of 130 patients who received HBOT. Thirteen male patients (13/121; 10.74%) and 2 female patients died (2/9; 22.22%). However, the difference between the groups was not significant (*p* = 0.278). Eight studies reported age data for 97 patients who received HBOT. Three patients younger than 60 years (3/51; 5.88%) died, whereas 9 patients older than 59 years (9/46; 19.56%) died. A trend towards a difference between the groups was observed (*p* = 0.062). In terms of comorbidities, no significant differences were observed between surviving and deceased patients with FG who received HBOT, including those with diabetes mellitus and alcohol use disorder. No study has explored the risk factors for mortality among patients with FG who underwent HBOT. The current studies include very few patients; thus, we cannot draw any conclusions.

Although the observed reduction in mortality with HBOT is promising, HBOT should be regarded strictly as an adjunct therapy and considered only after urgent and thorough debridement, not as a primary intervention in the acute setting. In addition, HBOT is limited by cost and availability and should be considered only as an adjunct therapy for selected high-risk patients without delaying surgery or broad-spectrum antibiotic therapy [48].

### 4.3. Limitations

This study has several limitations. The pronounced heterogeneity observed across the studies included in the analysis can be attributed to the diverse attributes present within the study populations. A wide spectrum of illness severity was observed among the included patients. Variations in the study design, conventional therapeutic strategies, and HBOT protocols were also noted. The temporal span of follow-up assessments exhibited considerable variability, and long-term follow-up data were unavailable for most patients. The retrospective nature of most of the included studies, coupled with the inherent characteristics of this methodological design, may introduce several sources of bias.

## 5. Conclusions

The evidence derived from this meta-analysis of retrospective studies indicates that the use of HBOT as an adjunct treatment tends to reduce mortality risk. Inherent biases associated with retrospective studies, such as patient selection, clinical severity, the HBOT protocol, and confounding factors, were observed. Given the infrequency of this condition, the limited accessibility of HBOT, and the intricate nature of FG, high-quality randomized controlled trials must be meticulously devised to determine the therapeutic efficacy of HBOT in patients with FG.

### Future Directions

1. What are the most effective hyperbaric oxygen treatment protocols (including pressure, duration, and frequency of sessions) for individuals with FG?
2. Which patient demographics obtain the greatest therapeutic benefit from HBOT?

## Figures and Tables

**Table 1:** Overview and characterization of fourteen included studies.

Study/Year	Study Design/Country	Participants	Administration of HBOT	Mortality	Authors Conclusion
Hollabaugh et al., 1996 [28]	RET/USA	26 patients (14 with HBOT and 12 without HBOT)	HBOT was offered to14 (54%) patients, The dive parameters were 90 min at 2.4 ATA and duration of therapy average 12 days.	Mortality: 7.1% (HBOT) vs. 41.6% (non-HBOT);	The clinical efficacy of HBOT was statistically proven to be a survival advantage for patients receiving adjuvant HBOT for FG.
Dahm et al., 2000 [29]	RET/USA	44 patients (38 with HBOT and 6 without HBOT)	Two to three sessions of 90 min at 2.5 to 3 ATA within the first 24 h, followed by re-evaluation of the patient	Mortality: 7.8% (HBOT) vs. 33.3% (non-HBOT);	There was a trend toward the improved survival of FG patients receiving HBOT.
Mindrup et al., 2005 [30]	RET/USA	42 patients (26 with HBOT and 16 without HBOT)	HBOT given 1–3 times (2.4–3 ATA, 30–90 min)	Mortality:26.9% (HBOT) vs. 12.5% (non-HBOT);	These data did not support routine HBOT in the treatment of Fournier’s gangrene and suggested that treatment may have been given to patients who were more ill.
Ayan et al., 2005 [31]	RET/ Turkey	41 patients (18 with HBOT and 23 without HBOT)	2.5 ATA for 90 min/day for 3 to 10 days	Mortality: 0% (HBOT) vs. 39.1% (non-HBOT);	Hyperbaric oxygen treatment is very effective in the treatment of FG.
Mehl et al., 2010 [32]	RET/Brazil	40 patients (26 with HBOT and 14 without HBOT)	120 min at a pressure of ATA that ranged from 2.0 to 2.8 ATA with a mean of 2.31 ATA. the number of cycles with an average of 12.5 cycles.	Mortality: 11.5% (HBOT) vs. 35.7% (non-HBOT);	Patients who underwent HBOT had a proportionately lower mortality rate when compared to those who did not receive it.
Li et al., 2015 [33]	RET/China	28 patients (16 with HBOT and 12 without HBOT)	HBOT was given twice a day for 5–7 days (2.5 ATA, 90–120 min each time, interval for 10 h)	Mortality: 12.5% (HBOT) vs. 33.3% (non-HBOT);	The effect of combining HBOT with conventional treatment offers considerable advantage in the treatment of FG.
Hung et al., 2016 [34]	RET/ Taiwan	60 patients (12 with HBOT and 48 without HBOT)	No exact specification	Mortality: 0% (HBOT) vs. 33.3% (non-HBOT);	Adjuvant HBOT led to higher survival rates.
Ferretti et al., 2017 [35]	RET/USA	20 patients (4 with HBOT and 16 without HBOT)	No exact specification	Mortality: 0% (HBOT) vs. 18.7% (non-HBOT);	HBOT is effective modern treatment modalities that are safe and feasible.
Anheuser et al., 2018 [36]	RET/ Germany	62 patients (17 with HBOT and 45 without HBOT)	No exact specification	Mortality: 0% (HBOT) vs. 4.4% (non-HBOT);	The positive influence of HBOT on the treatment of FG is going to be difficult to measure.
Gülşn et al., 2019 [37]	Case series /Turkey	14 patients (8 with HBOT and 6 without HBOT)	No exact specification	Mortality: 0% (HBOT) vs. 16.6% (non-HBOT);	HBOT was used in eight of our patients. The use of HBOT treatment may have played a role in our low mortality rate.
Creta et al., 2020 [38]	RET/Italy	161 patients (72 with HBOT and 89 without HBOT)	HBOT given twice a day for 4–7 days (2.4–2.8 ATA, for 50–90 min)	Mortality: 19.4% (HBOT) vs. 35.9% (non-HBOT);	HBOT, as adjunctive treatment in patients with FG, significantly reduces disease-related mortality.
Michalczyk et al., 2021 [39]	RET/ Poland	35 patients (13 with HBOT and 22 without HBOT)	No exact specification	Mortality: 0% (HBOT) vs. 18.1% (non-HBOT);	HBOT showed advanced wound healing with a high efficiency rate.
Feres et al., 2021 [22]	RET/Brazil	197 patients (79 with HBOT and 118 without HBOT)	The sessions were performed daily, for 2 h, with a pressure of 2.4 ATA. The protocol used was 15 consecutive sessions of HBOT.	Mortality: 2.5% (HBOT) vs. 28.8% (non-HBOT);	The use of adjuvant HBOT in combination with classical treatment was associated with reduced mortality.
Tutino et al., 2022 [40]	RET/Italy	23 patients (13 with HBOT and 10 without HBOT)	HBOT was offered to 13 (56.5%) patients using a scheduled session of 60 min daily	Mortality: 15.3% (HBOT) vs. 10.0% (non-HBOT);	HBOT did not offer an improvement in mortality when added to surgical debridement plus antibiotic therapy.

Foot notes: RET: retrospective study; USA: United States of America; HBOT: hyperbaric oxygen therapy; ATA: atmosphere absolute; vs.: versus; FG: Fournier’s gangrene.

**Table 2:** Risk bias of fourteen included studies.

Author/Year	Confounding	Selection	Interventions Classification	Interventions Deviations	Missing Data	Measurement of Outcomes	Selective Results
Hollabaugh et al., 1996 [28]	serious risk	serious risk	high risk	high risk	serious risk	high risk	serious risk
Dahm et al., 2000 [29]	moderate risk	low risk	lows risk	moderate risk	moderate risk	low risk	moderate risk
Mindrup et al., 2005 [30]	high risk	high risk	serious risk	high risk	high risk	moderate risk	high risk
Ayan et al., 2005 [31]	serious risk	serious risk	serious risk	serious risk	high risk	high risk	serious risk
Mehl et al., 2010 [32]	moderate risk	low risk	lows risk	moderate risk	moderate risk	low risk	moderate risk
Li C et al., 2015 [33]	serious risk	serious risk	high risk	serious risk	serious risk	high risk	serious risk
Hung et al., 2016 [34]	moderate risk	Low risk	lows risk	moderate risk	moderate risk	low risk	moderate risk
Ferretti et al., 2017 [35]	serious risk	serious risk	serious risk	serious risk	serious risk	serious risk	serious risk
Anheuser et al., 2018 [36]	low risk	low risk	moderate risk	moderate risk	low risk	low risk	low risk
Gülşen et al., 2019 [37]	serious risk	serious risk	serious risk	serious risk	serious risk	serious risk	serious risk
Creta et al., 2020 [38]	low risk	low risk	moderate risk	low risk	low risk	low risk	low risk
Michalczyk et al., 2021 [39]	high risk	high risk	serious risk	high risk	high risk	high risk	high risk
Feres et al., 2021 [22]	low risk	low risk	low risk	low risk	low risk	low risk	low risk
Tutino et al., 2022 [40]	high risk	serious risk	high risk	high risk	serious risk	serious risk	serious risk

**Table 3:** The mortality risk factors among patients with Fournier’s gangrene who underwent hyperbaric oxygen therapy.

Risk Factors	Number of Mortality	Number of Survival	Fisher’s Exact Test
**Age**			
<60 years old	3 (5.88%)	48	0.062
≥60 years old	9 (19.56%)	37	
**Gender**			
male	13 (10.74%)	108	0.278
female	2 (22.22%)	7	
**FGSI**			
≤9	0 (0%)	15	0.091
>9	2 (28.57%)	5	
**Comorbidities**			
DM	7 (16.67%)	35	0.561
No DM	6 (12.0%)	44	
Alcoholism	0 (0%)	8	1.0
No alcoholism	1 (4.54%)	21	

Foot notes: FGSI: Fournier’s gangrene Scoring Index; DM: diabetes mellitus.

## Data Availability

The original contributions presented in this study are included in the article/Supplementary Material. Further inquiries can be directed to the corresponding author.

## Supplementary Materials

The following supporting information can be downloaded at: https://www.mdpi.com/article/10.3390/medicina62061199/s1. Table S1: PRISMA 2020 Checklist. Reference [51] is cited in the Supplementary Materials.
