# Supplementary material for: Hyperbaric Oxygen Therapy Is Associated with Lower Mortality in Patients with Fournier’s Gangrene: A Meta-Analysis
Source: Medicina (Kaunas). 2026 Jun 22;62(6):1199. doi: 10.3390/medicina62061199 (PMC13303413; doi:10.3390/medicina62061199)
Supplement: Supplementary file 1 [file medicina-62-01199-s001.zip › medicina-4370929-supplementary.pdf]

PRISMA 2020 Checklist

Section and Topic	Item #	Checklist item	Location where item is reported
**TITLE**			
Title		Hyperbaric oxygen therapy reduces mortality in patients with Fournier's gangrene: A meta-analysis	2-3
**ABSTRACT**			
Abstract	2	Background and Objectives: The therapeutic management of Fournier's gangrene involves broad-spectrum antibiotics accompanied by sufficient surgical debridement; however, the global mortality remains high. Hyperbaric oxygen therapy has been suggested as an adjunctive treatment modality to reduce mortality, but its efficacy remains a topic of considerable debate. Consequently, this meta-analysis was conducted to assess the mortality rate associated with hyperbaric oxygen therapy for Fournier's gangrene.<br>Materials and Methods: We assessed the impact of hyperbaric oxygen therapy in patients with Fournier's gangrene. A search was meticulously conducted across various databases focusing on parameters such as population, intervention, control, and mortality rates.<br>Results: Fourteen studies were included in the analytical review. The aggregate number of patients evaluated in this meta-analysis was 793, comprising 356 in the hyperbaric oxygen therapy cohort and 437 in the non-hyperbaric oxygen therapy cohort. Compared with 27.45% in the control cohort, the mortality rate in the hyperbaric oxygen therapy cohort was 9.55%, A statistically significant disparity in mortality was evident between patients diagnosed with Fournier's gangrene who underwent conventional therapy with hyperbaric oxygen therapy and those receiving only conventional therapy (RR=0.33; 95% CI 0.23–0.48; $p < 0.001$).<br>Conclusion: Hyperbaric oxygen therapy may serve as an adjunctive intervention to mitigate the heightened risk of mortality among individuals with Fournier's gangrene. Methodologically rigorous randomized controlled trials must be conducted to determine the appropriate dosage and therapeutic efficacy of hyperbaric oxygen therapy in patients with Fournier's gangrene and identify specific patient cohorts poised to derive benefit from such treatment modalities.	25-45
**INTRODUCTION**			
Rationale	3	Fournier's gangrene (FG) is an uncommon, swiftly advancing necrotizing soft tissue infection characterized as necrotizing fasciitis affecting the scrotal, perineal, anal, and genital anatomical regions. The management of FG is a multifaceted challenge that requires an interdisciplinary methodology. It is predicated on three fundamental interventions: prompt surgical debridement, empirical administration of broad-spectrum antibiotics, and intensive supportive care.<br>A study by Eke N et al. reported 1726 FG cases with a mortality rate of 16% in 2000. A study by Sorensen MD et al. reported 1641 FG cases in the US population, with a mortality rate of 7.5% in 2016. However, the global mortality of Fournier's gangrene has remained high, ranging from 20–40%. Shet et al. conducted a thorough analysis of mortality risk factors in patients with FG, reporting a mortality rate of 20.4% among a cohort of 3646 patients. Female sex, the presence of comorbidities, anatomic distribution, the development of sepsis, and fungal infection have been shown to increase the risk of mortality.<br>FG continues to be associated with elevated mortality rates. This phenomenon can be attributed to inadequate local blood circulation in patients, which leads to infection and vascular impairment, thereby prolonging the healing process. Identifying an adjunctive therapeutic approach to conventional treatment modalities is imperative, as it may substantially increase survival rates and mitigate mortality risk in patients with necrotizing fasciitis. Hyperbaric oxygen therapy (HBOT) represents a therapeutic intervention that involves inhaling pressurized 100% oxygen in a sealed chamber operating at pressures exceeding atmospheric levels and is utilized as an adjunctive treatment modality for FG; Several studies have reported that HBOT contributes to a significant reduction in mortality among patients with Fournier's gangrene. The 2024 European Association of Urology Guidelines on Urological Infections recommend that adjunctive treatments for Fournier's gangrene be administered only within the framework of clinical trials. There remains a lack of consensus regarding the efficacy of adjunctive therapy with HBOT in FG, particularly in relation to its potential to reduce mortality among affected patients.	55-117
Objectives	4	The purpose of this meta-analysis was to investigate the effect of HBOT on mortality in FG patients.	117-118
**METHODS**			
Eligibility criteria	5	**Population:** We included patients with Fournier's gangrene. Data were manually extracted from the eligible full-text articles. The extracted items included the authors, year of publication, study design, total number of HBOT patients, total number of Non-HBOT group patients and mortality rate. **Intervention**: The studies were considered eligible for inclusion only if they directly compared the mortality rate of HBOT patients and Non-	129-136

Section and Topic	Item #	Checklist item	Location where item is reported
		HBOT group patients. **Comparison:** HBOT group patients versus Non-HBOT group patients. **Outcome:** 1.Hospital mortality. 2. The risk factors for mortality among patients with FG receiving HBOT.	
Information sources	6	We looked through electronic databases, such as Pubmed, Cochrane Library, and Web of Science, from the time of January 1, 1990 until February 28, 2026.to find pertinent published trials, and meta-analyses or systematic reviews on the topic.	121-125
Search strategy	7	The search terms include Fournier Gangrene",“Penile necrotizing fasciitis”, “Hyperbaric oxygen”,“Hyperbaric oxygen therapy”, and“Hyperbaric oxygen treatment” to find pertinent published trials, and meta-analyses or systematic reviews on the topic. Previously published systematic reviews and meta-analyses were reviewed to identify any additional studies that may have been missed in the primary literature search. No restrictions on the usage of English were applied..	121-127
Selection process	8	Articles containing relevant terms in each database were identified and imported into Endnote Library for the deletion of duplicate records. After excluding duplicates, all studies were reviewed by reading the title and/or abstract to identify irrelevant studies. To determine the eligibility of the identified trial reports, two of the authors independently screened the titles and abstracts. After excluding irrelevant studies, all of the relevant articles were reviewed by reading the full text to determine eligible trial reports. (Fig 1)	138-140
Data collection process	9	To determine the eligibility of the identified trial reports, two of the authors independently screened the titles and abstracts. After excluding irrelevant studies, all of the relevant articles were reviewed by reading the full text to determine eligible trial reports. Any inconsistencies or conflicts pertaining to data extraction were deliberated upon and resolved by a third investigator, as deemed necessary.	140-143
Data items	10a	Eligible outcome is mortality rate.	143
	10b	The extracted items included the authors, year of publication, study design, number of participants in each treatment group, and mortality rate, all of which were derived from studies that met the established inclusion criteria. We also explored the risk factors for mortality among patients with FG receiving HBOT.	140-141
Study risk of bias assessment	11	The Risk of Bias in Nonrandomized Studies of Interventions (ROBINS-I) tool was used to evaluate the observational studies	145-146
Effect measures	12	Statistical analyses were conducted utilizing RevMan 5.4 and Cochrane Review Manager software. Data were entered into the Cochrane Review Manager software RevMan 5. Differences were expressed as risk ratios (RRs) with 95% confidence intervals (CIs) for dichotomous outcomes. The significance of the pooled ratios was determined by the Z test, and a P value less than 0.05 was considered statistically significant. Furthermore, Fisher’s exact test was employed to investigate mortality risk factors among FG patients receiving HBOT.	146-157
Synthesis methods	13a	We use the Forest plots to tabulate the study intervention characteristics and compare against the planned groups for each synthesis.	146-157
	13b	There is no converting reported statistics to required statistics, transforming effect estimates, or imputing missing summary data. Therefore, we do not prepare the data collected from studies.	146-157
	13c	Data were entered into the Cochrane Review Manager software RevMan 5. We use Forest plot to displays year of publication, size of the effect estimates, study weight, and confidence intervals of each study.	146-157
	13d	The I^2 test was used to assess the proportion of statistical heterogeneity and I^2 more than 50% was considered significant among the studies. The Q-statistic test was used to define the degree of heterogeneity. A P value less than 0.10 for the Q-test was considered significant among the studies. The fixed-effects model was used when the effects were assumed to be homogenous, while the random-effects model was used when they were heterogeneous. Publication bias was assessed by examining the funnel plot. All analyses were entered into the Cochrane Review Manager software RevMan 5.	146-157
	13e	Subgroup analysis or meta-regression was not performed in this study.	NA
	13f	We conducted a sensitivity analysis by systematically removing each study and assess the impact of the study quality on the effect estimates.	146-157
Reporting bias	14	We planned to generate funnel plots for meta-analyses.(Figure 3)	

Section and Topic	Item #	Checklist item	Location where item is reported
assessment			
Certainty assessment	15	Quality of the evidence was ranked based on the risk of bias according to the Grading of Recommendations Assessment, Development and Evaluation (GRADE) approach at the outcome level.	NA
**RESULTS**			
Study selection	16a	14 studies were included in the meta-analysis. A total of 793 patients were analyzed in this meta-analysis, comprising 356 in the HBOT group and 437 in the non-HBOT group. The intervention groups in each study were given different doses of HBOT. The overall mortality rate in the hyperbaric oxygen group was 9.55%, compared with 27.45% in the control group. There was a significant difference in mortality between patients receiving conventional therapy with HBOT and those receiving only conventional therapy alone (RR=0.33; 95% CI 0.23–0.48; p < 0.001) (Figure 2).	163-182
	16b	Ten studies reported risk factors for mortality among patients with FG who underwent HBOT (Table 3).	546-550
Study characteristics	17	The overview and characteristics of the 14 included studies are shown in Table 1.	532-538
Risk of bias in studies	18	All studies had a high risk of bias (Table 2).	543-545
Results of individual studies	19	For all outcomes, present, for each study: Figure 1 to Figure 3 and table 3.	495-521 & 546-550
Results of syntheses	20a	14 studies were included in the meta-analysis. The main characteristics of the included studies are shown in Table 1. All studies had a high risk of bias (Table 2). Ten studies reported risk factors for mortality among patients with FG who underwent HBOT (Table 3).	171-175
	20b	The intervention groups in each study were given different doses of HBOT. The overall mortality rate in the hyperbaric oxygen group was 9.55%, compared with 27.45% in the control group. There was a significant difference in mortality between patients receiving conventional therapy with HBOT and those receiving only conventional therapy alone (RR=0.33; 95% CI 0.23–0.48; p < 0.001) (Figure 2 and Figure 3)	176-182
	20c	Two studies reported FGSI scores for 22 patients receiving HBOT. No deaths occurred among patients with an FGSI score ≤ 9, whereas 2 patients (2/7; 28.5%) with an FGSI score more than 9 died. However, there was no significant difference between these groups (p=0.091). Nine studies reported the sex distribution of 130 patients receiving HBOT. Thirteen male patients (13/121; 10.74%) and 2 female patients died (2/9; 22.22%). However, there was no significant difference between the groups (p=0.278). Eight studies reported age data for 97 patients receiving HBOT. Three patients younger than 60 years (3/51; 5.88%) died, compared with 9 patients over 59 years (9/46; 19.56%). There was a trend towards a difference between the groups (P=0.062). In terms of comorbidities, no significant differences were observed between surviving and deceased in patients with FG who received HBOT, including those with diabetes mellitus and alcoholism	241-251
	20d	For the sensitivity analysis, no study was eliminated from the original study pool.	NA
Reporting biases	21	We use funnel plot to assess of risk of bias due to missing results (Figure3).	146-157
Certainty of evidence	22	Our findings demonstrated a significant reduction in mortality among patients receiving conventional therapy with HBOT compared with those receiving conventional therapy alone. Compared with 27.45% in the control group, the overall mortality rate in the HBOT group was 9.55%. Meta-analysis revealed a relative risk of mortality of 0.33, indicating that patients treated with HBOT had a 67% lower risk of death than controls.	176-182
**DISCUSSION**			
Discussion	23a	HBOT accelerates the healing process and has a bactericidal effect on anaerobic infections caused by either aerobic or anaerobic bacteria. However, the optimal duration and frequency of treatment for necrotizing soft tissue infections remain to be definitively determined, and standardized protocols are lacking. The 10th European Consensus Conference (2017) on Hyperbaric Medicine established the following consensus-based recommendations regarding standard practices in hyperbaric medicine: 1. HBOT is advocated for the management of anaerobic or mixed bacterial infections; 2. HBOT should be employed for the treatment of necrotizing soft tissue infections in any anatomical location, with particular emphasis on FG; and 3. HBOT must be integrated with prompt surgical intervention and broad-spectrum antibiotic	184-208

Section and Topic	Item #	Checklist item	Location where item is reported
		therapy tailored to address prevalent anaerobic and aerobic bacterial pathogens [49]. Some studies reported that HBOT did not reduce mortality in patients with FG. Mindrup et al. reported that patients who underwent HBOT had a higher mortality rate—12.5% in the non-HBOT group vs. 26.9% in the HBOT group [31]. This increase in mortality may be attributable to selection bias, as more severely ill patients may have necessitated HBOT. In addition, the median follow-up in this study was 4.2 years, significantly longer than in other studies. The severity of FG and longer follow-up durations are important factors in determining the real outcomes and prognosis of patients with FG. In the study by Tutino et al., a mortality rate of 15.38% was reported in the hyperbaric oxygen group, compared with 10.0% in the conventional therapy group, indicating that HBOT did not improve the survival rate of patients with FG [42]. A higher percentage of female patients with FG (7/23; 30.4%) was included in this study, including two female patients who died after receiving HBOT. Mortality rates were notably higher among females (29.76%) than among males (20.48%) in the study of Shet P et al. [15]. The higher percentage of female patients with FG may be the reason HBOT did not improve the survival rate of patients with FG in Tutino's study.	
	23b	Several meta-analyses and systematic reviews have investigated the impact of HBOT in patients with FG and have demonstrated that mortality rates were markedly lower in the HBOT group than in the non-HBOT group. The comprehensive systematic review conducted by Schneidewind L et al. included five studies that collectively involved 319 patients diagnosed with FG, of whom 145 were administered HBOT. This resulted in a mortality rate of 16.6% in the HBOT cohort and 25.9% in the non-HBOT cohort [50]. The systematic review by Shet P. et al. included 57 studies, comprising 3646 patients with FG with an overall mortality rate of 20.41%. Among these, 42 patients received HBOT and had a lower mortality rate of 11.9% [15]. The meta-analysis by Raizandha MA et al. included 10 retrospective studies from 1998 to 2021, involving 269 patients who received HBOT. Patients in the HBOT group had significantly lower mortality than those in the non-HBOT group (odds ratio 0.29, p = 0.005) [51]. The meta-analysis by Patel A et al. (2026) included 9 retrospective studies and 4 case series, with 229 patients receiving HBOT. Patients in the HBOT group had significantly lower mortality (11.5%) compared with the non-HBOT group (26.0%) (RR=0.43, 95% CI 0.21–0.86). The authors suggested that HBOT may reduce mortality in patients with FG and could be considered an adjunctive therapy [52]. An umbrella review by Mussgens C et al. (2026) included seven systematic review articles and concluded that HBOT may reduce mortality, although the evidence remains limited [53]. The primary objective of this review was to assess the impact of HBOT on mortality in patients with FG. This meta-analysis included 14 retrospective studies from January 1990 to February 2026, of which 356 patients received HBOT. Our findings demonstrated a significant reduction in mortality among patients receiving conventional therapy with HBOT compared with those receiving conventional therapy alone. Compared with 27.45% in the control group, the overall mortality rate in the HBOT group was 9.55%. Meta-analysis revealed a relative risk of mortality of 0.33, indicating that patients treated with HBOT had a 67% lower risk of death than controls.	209-233
	23c	A systematic review by Shet P. et al. explored risk factors for mortality among patients with FG and found that the mean FGSI score for nonsurvivors was 10.09 versus 4.33 for survivors, with studies consistently showing that a cut-off score of nine or more was strongly associated with mortality (OR=4.11, p = 0.024). The mean age of those who died was 61.27 years, whereas the mean age of survivors was 53.21 years. Compared with males (20.48%), the mortality rate was notably higher among females (29.76%) [15]. In the current meta-analysis, two studies reported FGSI scores for 22 patients receiving HBOT. No deaths occurred among patients with an FGSI score ≤ 9, whereas 2 patients (2/7; 28.5%) with an FGSI score more than 9 died. However, there was no significant difference between these groups (p=0.091). Nine studies reported the sex distribution of 130 patients receiving HBOT. Thirteen male patients (13/121; 10.74%) and 2 female patients died (2/9; 22.22%). However, there was no significant difference between the groups (p=0.278). Eight studies reported age data for 97 patients receiving HBOT. Three patients younger than 60 years (3/51; 5.88%) died, compared with 9 patients over 59 years (9/46; 19.56%). There was a trend towards a difference between the groups (P=0.062). In terms of comorbidities, no significant differences were observed between surviving and deceased in patients with FG who received HBOT, including those with diabetes mellitus and alcoholism. No study has explored the risk factors for mortality among patients with FG who underwent HBOT. Current studies include very few patients; thus, we cannot draw any conclusions.	234-253
	23d	Although the observed reduction in mortality with HBOT is promising, it should be regarded strictly as an adjunctive therapy, considered only after urgent and thorough debridement, not as a primary intervention in the acute setting. In addition, HBOT is limited by cost and availability and should be considered only as an adjunctive therapy for selected high-risk patients without delaying surgery and broad-spectrum antibiotic therapy [50].	254-258
OTHER INFORMATION			

PRISMA 2020 Checklist

Section and Topic	Item #	Checklist item	Location where item is reported
Registration and protocol	24a	register name: Chienhsiu Huang; Institutional Review Board Statement: The meta-analysis was registered at the Prospero international prospective register of systematic reviews (registration No. CRD420261369219). (registration date: April 14,2026)	158-161
	24b	Protocol was reported in the Prospero international prospective register of systematic reviews (registration No. CRD420261369219).	NA
	24c	NA	
Support	25	non-financial support for the review	289
Competing interests	26	No competing interests of review authors.	293
Availability of data, code and other materials	27	Data described in the manuscript, code book, and analytic code will be made available upon request pending and raw extraction tables are accessible.	291-292

From: Page MJ, McKenzie JE, Bossuyt PM, Boutron I, Hoffmann TC, Mulrow CD, et al. The PRISMA 2020 statement: an updated guideline for reporting systematic reviews. BMJ 2021;372:n71. doi: 10.1136/bmj.n71

For more information, visit: http://www.prisma-statement.org/
